# Fluid and Salt Balance Are Things We Often Overlook: Could Our Understanding of Fluid Dynamics Change How We Tackle Heart failure?

**DOI:** 10.1016/j.mayocpiqo.2024.02.001

**Published:** 2024-03-06

**Authors:** Shriya Sharma, Rohan Goswami

**Affiliations:** Division of Heart Failure and Transplant, Mayo Clinic, Jacksonville, FL

Heart failure (HF) mechanisms involve reduced cardiac output, elevated systemic venous pressures, and compromised renal perfusion, collectively triggering the sympathetic nervous system (SNS) and the renin-angiotensin-aldosterone system. Physicians dealing with a saturated clinic with a plethora of medication-noncompliant patients in our current system are stymied in providing further education beyond the importance of adhering to prescribed therapies. But, is this the best first approach?

We know that managing HF treatment involves addressing patients with refractory acute decompensated HF and stabilizing those with chronic HF. Beyond medications targeting the neurohormonal system, maintaining an intricate balance of salt and fluid plays a pivotal role in sustaining clinical equilibrium and safeguarding renal function.^1^^,^^2^

Managing fluid overload in patients with HF presents a delicate challenge because the margin between dehydration and volume overload is narrow. The principal objective in such scenarios is to restore fluid balance while safeguarding cardiac performance from further deterioration. Avoid falling off the starling curve. This becomes especially complex in right HF, where low cardiac output, venous congestion, and diminished preload lead to progressive renal retention of salt and water, exacerbating extracellular expansion and myocardial distension.^3^

Using the clinical examination and remote monitoring technologies and helping patients understand their symptoms (which may not just be peripheral edema) are key for patients owning their health care and improving their outcomes.

Sodium and fluid restriction are fundamental nonpharmacologic approaches extensively employed in managing acute decompensated HF. Although their therapeutic efficacy lacks evidence, these measures are recommended in textbooks and clinical practice guidelines, and they work. Despite remarkable advancements in pharmacologic and device-based therapies, HF remains the most prevalent reason for hospital admissions among individuals older than 65 years.^1^

Restricting dietary sodium is commonly advised as a routine self-care practice for patients with HF, and it is a mandated component of HF hospital discharge instructions. On average, Americans typically consume 3400-3700 mg of sodium daily. Recent recommendations from the American Heart Association propose a population-wide sodium restriction to less than 1500 mg/d, primarily based on data from cohort studies in hypertensive individuals without HF. The Heart Failure Society of America suggests a daily sodium intake of 2-3 g for all patients with HF, with further restriction to less than 2 g for those with “moderate to severe HF.” The 2012 European Society of Cardiology HF Guidelines do not provide specific recommendations on sodium intake for chronic HF management.^4^

The existing guidelines vary, reflecting the limited data on sodium restriction in HF. Although most observational studies support the idea that low sodium intake benefits HF outcomes, a few controlled trials, although challenging to interpret, suggest that strict sodium restriction might be harmful in some patients with HF. Dietary sodium restriction can have physiological consequences, potentially contributing to adverse outcomes in HF, particularly when combined with unmonitored diuretic therapy and fluid restriction, especially in the era of novel therapies such as SGLT2 inhibitors.^5^

Observational studies support that dietary sodium restriction improves HF outcomes, particularly in patients with higher disease severity and symptom burden. Resolving whether and how much to restrict sodium to prevent HF-associated complications may hinge on personalized medication plans to mitigate the risks of hyponatremia. Dietary sodium restriction, especially when coupled with the Dietary Approaches to Stop Hypertension eating plan, has been shown to lower blood pressure, reduce oxidative stress, and enhance vascular function in salt-sensitive individuals.

Congestion exacerbates diuretic resistance by affecting absorption, stimulating the SNS, and activating the renin-angiotensin-aldosterone system. Additionally, compromised liver function in cases of right HF contributes to hypoalbuminemia, intensifying resistance to commonly prescribed diuretics. This progressive cardiorenal dysfunction and diuretic resistance culminate in unfavorable clinical outcomes despite escalating doses of loop diuretics. In decompensated HF and congestion, the primary driver of diuretic resistance arises from elevated renal vein pressure, leading to alterations in pressure gradients and impeding renal function.

Individualized recommendations for fluid restriction and salt limitation are key to optimizing the outcomes of guideline-directed medical therapy in patients with acute on chronic decompensation. As outlined in the Evaluation Study of Congestive Heart Failure and Pulmonary Artery Catheterization Effectiveness (ESCAPE) trial and subsequent analysis, increased intracardiac filling pressures are associated with worse outcomes. Because to the renin-angiotensin-aldosterone cascade of sodium and water retention, temporary sodium restriction in ambulatory outpatients may limit hospitalization (eg, New York Heart Association 2, American Heart Association C, and Stevenson profile B). Similarly, achieving euvolemia in patients may allow for a decrease in sympathetic tone and better tolerance of optimal doses of guideline-directed medical therapy, improving the quality and quantity of life, while reducing readmission and improving functional status.^6^^,^^7^

Let us consider the systemic effects of managing sodium and fluid intake (not just water intake). We begin to appreciate the downstream effect of less myocardial stress—less arrhythmia burden, improved filling pressures, better biventricular synchronicity, and geometry—all things proven in device and medication trials but, now, not requiring an invasive medical procedure or adding “another pill” to make an impact.

Accurate quantification of sodium intake continues to be a hurdle. However, patient and provider awareness results in understanding and awareness of what patients are actually consuming. If asked about your exercise frequency, you would not say what you know it is. You will answer what you want it to be. The same goes for our patient’s salt and fluid intake, medication compliance, and exercise regimens.

Understanding the pluripotent impact of our current pillars of HF therapy aligns with one singular principle—reduction in sympathetic activation and optimization of natriuresis. A key foundation of these principles is that patients with HF experience reduced renal perfusion. This creates an underlying presence of SNS activity that compensates for diminished cardiac output by increasing sodium and water retention in the kidney. Downstream effects of increased vasoconstriction, elevation in left ventricular afterload, and worsening cardiac output create a vicious cycle. Innovating our approach to critically address the crux of this issue—salt and water retention—especially in the United States, may jump-start the decrease in sympathetic tone by driving down ventricular filling pressures, which are directly linked to worsening HF symptoms. Unfortunately, current clinical evidence supporting the practice of dietary sodium restriction is inconsistent, and the focus on increasingly complicated medication regimens prevails.

We provide patient education and offer simple practical solutions:

1. You should limit your fluid intake to 50 oz (1500 mL) per day, including the following:
  a. All round fruit are 10 ounces per serving (eg, apple, oranges, peaches, pears, plums, etc)
  b. All berries and grapes are 1 ounce each (eg, 10 grapes = 10 oz of fluid)
  c. All melon/pineapple are 12 ounces per serving, a serving is 3 slices or 1 bowl of chopped fruit
2. You should limit your salt intake to 2000 mg/d
  a. Frozen foods still have salt, make sure to read the package labeling for salt content and choose options that state “preservative free”
  b. Avoid/stop all canned foods to reduce salt intake
  c. When choosing salt “alternatives,” such as Mrs. Dash, verify that these are okay to use with your provider first

Evidence-based data from trials in the early 2000s (eg, ESCAPE, Paradigm HF, and Dapagliflozin in Patients with Heart Failure and Reduced Ejection Fraction) clearly outline the benefit of both hospitalized and nonhospitalized patients maintaining a euvolemic state (eg, right atrial pressure, <10 mm Hg; echocardiography-based IV collapse); however, randomized clinical trials to associate this directly with sodium are improbable and unlikely in the current era of advanced HF therapies. Corollaries to this, however, have come to pass. As we evaluate the data from trials supporting invasive pulmonary arterial catheter pressure monitoring and reduction in HF readmissions—the key take-home from multiple analysis, including a prospective randomized trial by Adamson et al^8^ shows that (1) patients with increased filling pressures were more likely to be readmitted; (2) increase in diuretic dose was related to frequent monitoring of pulmonary arterial pressures; and (3) equivalent doses of neurohormonal antagonists were seen in the majority of patients. This supports the fact that increased filling pressures—due to more salt and fluid intake—was mitigated by increased diuresis (more salt and fluid removal). Limiting intake may mitigate the need for such aggressive strategies that could prove detrimental to renal function, systemic blood pressures, and worsen extracardiac symptoms such as cramping or dizziness. This links directly to the current methods in place in HF clinics: frequent patient touch points to assess high-risk patients, virtual or in-person nutrition, pharmacy and cardiac rehabilitation consultations, and support groups for patients to discuss strategies that work well. The balance of increased resource utilization with frequent clinic visits or noninvasive data monitoring are potentially balanced by improved patient quality of life and less readmission, especially in the era of publicly reported outcomes and ties to institutional reimbursement.

We are all aware of the innovation of medicine and technology—it is time to return to a way of dealing with HF, where simple things become powerful tools for changing lives.

## Potential Competing Interests

The authors declared no potential conflicts of interest with respect to the research, authorship, and/or publication of this article.
